# Pirfenidone ameliorates alcohol-induced promotion of breast cancer in mice

**DOI:** 10.3389/fonc.2024.1351839

**Published:** 2024-03-25

**Authors:** Hui Li, Mei Xu, Danlei Chen, Wen Wen, Jia Luo

**Affiliations:** ^1^ Department of Pathology, University of Iowa Carver College of Medicine, Iowa City, IA, United States; ^2^ Department of Pharmacology and Nutritional Sciences, University of Kentucky College of Medicine, Lexington, KY, United States; ^3^ Department of Hepatobiliary Surgery, The First Affiliated Hospital of USTC, Division of Life Sciences and Medicine, University of Science and Technology of China, Hefei, Anhui, China; ^4^ Iowa City VA Health Care System, Iowa City, IA, United States

**Keywords:** alcohol use disorders, cancer stem cells, drug therapy, mammary tumors, metastasis

## Abstract

**Purpose:**

Alcohol consumption increases the risk of breast cancer and promotes cancer progression. Alcohol exposure could affect both processes of the mammary carcinogenesis, namely, the cell transformation and onset of tumorigenesis as well as cancer aggressiveness including metastasis and drug resistance/recurrence. However, the cellular and molecular mechanisms underlying alcohol tumor promotion remain unclear. There are four members of the mammalian p38 mitogen-activated protein kinase (MAPK) family, namely, p38α, p38β, p38γ and p38δ. We have previously demonstrated alcohol exposure selectively activated p38γ MAPK in breast cancer cells *in vitro* and *in vivo*. Pirfenidone (PFD), an antifibrotic compound approved for the treatment of idiopathic pulmonary fibrosis, is also a pharmacological inhibitor of p38γ MAPK. This study aimed to determine whether PFD is useful to inhibit alcohol-induced promotion of breast cancer.

**Methods:**

Female adolescent (5 weeks) MMTV-Wnt1 mice were exposed to alcohol with a liquid diet containing 6.7% ethanol. Some mice received intraperitoneal (IP) injection of PFD (100 mg/kg) every other day. After that, the effects of alcohol and PFD on mammary tumorigenesis and metastasis were examined.

**Results:**

Alcohol promoted the progression of mammary tumors in adolescent MMTV-Wnt1 mice. Treatment of PFD blocked tumor growth and alcohol-promoted metastasis. It also significantly inhibited alcohol-induced tumorsphere formation and cancer stem cell (CSC) population.

**Conclusion:**

PFD inhibited mammary tumor growth and alcohol-promoted metastasis. Since PFD is an FDA-approved drug, the current findings may be helpful to re-purpose its application in treating aggressive breast cancer and alcohol-promoted mammary tumor progression.

## Introduction

Both epidemiological and experimental studies indicate that alcohol consumption increases the risk of breast cancer and promotes cancer progression (1–6). In addition to the promotion of the tumorigenesis, alcohol may also enhance the growth of existing breast tumors and increase the aggressiveness of breast cancer cells to invade and metastasize (5). However, the cellular and molecular mechanisms underlying alcohol tumor promotion remain unclear. We have previously demonstrated that alcohol exposure selectively activated p38γ MAPK without affecting other p38 MAPK isoforms in breast cancer cells *in vitro* and *in vivo* (7–9). p38γ MAPK is a member of p38 MAPK family which contains four isoforms p38α, p38β, p38γ, and p38δ. Although p38γ MAPK has been less investigated, it has unique functions particularly in the regulation of cell cycle progression, mobility, migration/invasion, cancer stem cells (CSCs), and epithelial-mesenchymal transition (EMT) (10, 11). The activation and over-expression of p38γ MAPK is associated with malignant tumors (11, 12). We have previously showed that alcohol activated p38γ MAPK, which may lead to increased CSCs and metastasis of breast cancer cells (7–9). Therefore, these results suggest that p38γ MAPK may play an important role in alcohol-induced promotion of breast cancers.

Pirfenidone (PFD) is a synthetic pyridone compound that is an FDA-approved drug for the treatment of idiopathic pulmonary fibrosis (IPF). IPF is a progressive and fatal lung disease of unknown etiology. The therapeutic effects of PFD may be mediated by its beneficial property of anti-TGF-β signaling, anti-inflammation and anti-oxidative stress (13). PFD has recently been identified as a pharmacological inhibitor of p38γ MAPK (14–16). Since p38γ MAPK plays an important role in the progression of breast cancer and alcohol-induced promotion of mammary tumor, we hypothesized that the inhibition of p38γ MAPK activation by PFD was able to ameliorate alcohol-induced promotion of breast cancer. To test this hypothesis, we used an animal model of spontaneous mammary tumor, MMTV-Wnt1 mice in which alcohol was shown to promote breast cancer (9). Since adolescent MMTV-Wnt1 mice are more sensitive to alcohol-induced mammary tumor promotion (9), we used adolescent mice for this study. Our results indicated PFD effectively inhibited alcohol-induced promotion of tumorsphere formation, CSC population, tumor growth, and metastasis. Since PFD is an FDA-approved drug, the current findings may be helpful to re-purpose its application.

## Materials and methods

### Breast cancer cell cultures

The cultures of mouse and human breast cancer cells have been previously described (10, 17). Human breast cancer cells BT474 cells were cultured in full RPMI medium with insulin and 10% fetal bovine serum (FBS). SKRB3 cells were cultured in IMEM with 10% FBS. Mice mammary adenocarcinoma cell line E0771 was provided by Dr. Enrico Mihich (Roswell Park Cancer Institute, Buffalo, NY) and maintained in DMEM media supplemented with 10% FBS, penicillin (100 U/ml)/streptomycin (100 U/ml) and 0.25 mg/ml amphotericin B at 37°C in a humidified air containing 5% CO_2_. Pirfenidone (PFD) was obtained from Selleckchem (S-7701; Houston, TX). BT474, SKBR3 and E0771 cells were used for this study because they are aggressive breast cancer cell lines with high expression of p38γ MAPK and suitable for further studies of the role of p38γ MAPK in EMT and CSCs in mouse xenograft models (10, 17). The concentrations of PFD were selected based on previous studies showing effectiveness in cell cultures (18, 19).

### Animals and treatment

FVB MMTV-Wnt1 [FVB.Cg-Tg (Wnt1)1Hev/J, #002934] mice were obtained from The Jackson Laboratories (Bar Harbor, ME), bred, and housed in a climate-controlled animal facility. All procedures were reviewed and approved by the Institutional Animal Care and Use Committee (IACUC) of the University of Kentucky and the University of Iowa. Only female mice were used for this study. For alcohol exposure, adolescent mice (5 weeks-old) were assigned into control and alcohol exposure groups. Adolescent mice were used for this study because our previous study indicated that adolescent MMTV-Wnt1 mice were more sensitive to alcohol-induced mammary tumor promotion than adult mice (9). Mice were exposed to alcohol by feeding with alcohol containing liquid diet (Cat #: F1258SP, Bio-Serv, Flemington, NJ), while control mice were feed with isocaloric liquid diet (Cat #: F1259SP, Bio-Serv, Flemington, NJ) in which maltose was used to substitute isocalorically for alcohol. The alcohol concentration in the diet increased as the following: week 1, 2% alcohol; week 2, 4% alcohol; weeks 3 and on: 6.7% alcohol. Diet was provided ad libitum for the experimental period. During the experimental period, body weights of mice were evaluated. No significant body weight difference was observed among these animals. To monitor tumorigenesis, mice were examined weekly after the initiation of alcohol exposure. Tumor development/growth was monitored weekly. Mice with tumors exceeding 20 mm maximum diameter were euthanized and evaluated for metastasis. Mice were euthanized by IP injection of ketamine/xylazine (≥ 160 mg/kg/20 mg/kg). Dissected mammary tumor tissues or mammary glands were either immediately dissociated or fixed for the following procedures. To determine the blood alcohol concentrations (BACs), the blood was collected one week after feeding with 6.7% alcohol diet. The BACs were determined using Alcohol Analyzer AM1 (Analox Instruments, MA), and the mean BAC was around 80 mg/dl. PFD was dissolved in DMSO at 100 mg/ml and intraperitoneal (IP) injected to animals at 100 mg/kg two days before alcohol exposure. Mice received PFD injection every other day. The concentration of PFD was selected was based on previous studies in mice showing the effectiveness in inhibiting tumor growth (18, 20). The recommended concentration of PFD for the treatment of IPF in human is 1800 mg/day (21).

### Analysis of tumor volume and metastasis

The volume of the tumors was measured as previously described (22): two perpendicular dimensions of tumors were measured with a dial caliper. The volume was calculated based on the formula: V = 0.5a x b^2^; a is the longest and b is the shortest dimension. Tumor metastasis was determined as previously described (9). Briefly, when tumors reached 20 mm maximum diameter, mice were sacrificed, and lung tissues were removed and fixed with 4% paraformaldehyde. The paraffin-embedded lung tissues were sectioned at a thickness of 5 μm. The Hematoxylin–Eosin (H&E)-stained sections were examined and photographed under a microscope.

### Dissociation of mouse mammary tumor cells, determination of CSC population and tumorsphere formation

Dissociation of mouse mammary tumor cells was performed using reagents and procedures provided by STEMCELL Technologies Inc (Cambridge, MA). Briefly, resected mammary tumors were minced and incubated in collagenase/hyaluronidase-containing dissociation solution at 37°C for 4 hours. Pellets were washed with HBSS and Ammonium Chloride solution followed by incubations with Trypsin and then Dispase. Cells were washed by HBSS containing 2% FBS and then filtered through a 40 µm cell strainer. After centrifuging, the single-cell suspensions were collected for next experiments. The breast cancer stem cells were identified by aldehyde dehydrogenase (ALDH) activity and Thy1+/CD24+ as previously described (7, 9, 23). Briefly, dissociated mouse mammary tumor cells (5x10^5^ cells) were incubated with ALDEFLUOR assay buffer containing ALDH substrate for 45 min at 37°C. Some cells were stained under the same condition with a specific ALDH inhibitor as a negative control. Cells were sorted using flow cytometry and analyzed using WINMDI software. ALDEFLUOR-positive cells were considered as the population of CSCs. Data were presented relative to control groups. For Thy1+/CD24+ staining, briefly, dissociated mouse mammary tumor cells were incubated with fluorescent conjugated CD24 or Thy1+ antibodies for 30 min on ice followed by 2 times of wash in PBS. Cells were then analyzed by flow cytometry. Propidium iodide (PI) was used to determine the live cells which were subjected to the analysis.

Tumor sphere formation was determined as previously described (9). Briefly, dissociated single mammary tumor cell suspension (1000 cells) from either control or alcohol-fed mice were plated on ultra-low attachment plates in full Essential 8™ basal medium without further alcohol exposure, and incubated at 37°C and 5% CO_2_ for 10 days. The ability of tumor cells to form spheres was determined manually and presented relative to control groups.

### Immunoblotting

Mammary tissues were collected, and proteins were extracted. Around 30–50 μg of extracted protein was used in immunoblots to examine the levels of total and phosphorylated p38γ MAPK. The nitrocellulose membranes were first probed with specific primary antibodies overnight at 4°C. The generation and usage of primary phosphospecific antibody against p38γ MAPK has been previously described (7). Anti-p38γ MAPK antibody was obtained from R & D Systems (Cat # AF1347, Minneapolis, MN). Anti-p38α MAPK antibody (Cat # 9218) and Anti-GAPDH antibody (Cat # 2118) were obtained from Cell Signaling Technology (Danvers, MA). Anti-phospho-p38α antibody (Cat # 09-272) was obtained from Sigma Aldrich (St. Louis, MO). Anti-phospho-p38γ antibody was customized synthesized as previously described (7). After washing with TBS containing 0.05% Tween-20 three times, the membranes were incubated with anti-rabbit or anti-mouse secondary antibodies (horseradish peroxidase-conjugated) for one hour at room temperature. Protein-specific signals were then detected with enhanced chemiluminescence substrate (GE Healthcare, Chalfont, Buckinghamshire, UK) using a Chemi™Doc imaging system (Bio-Rad 215 Laboratories, Hercules, CA) and then quantified with the software of Image lab 5.2 (Bio-Rad Laboratories, Hercules, CA).

### MTT assay

To examine cell metabolic activity, 3-(4, 5-dimethyl-thiazol-2-yl)-2, 5-diphenyltetrazolium bromide (MTT) assay was used as previously described (8). Cells were seeded on 96-well plates at 2x10^3^ cells per well. At times indicated, MTT was added to each well at the final concentration of 500 µg/ml and incubated at 37°C for 2 hours. After the incubation, media were carefully removed and 100 µl DMSO was added to each well to dissolve the MTT formazan. Plates were read using the Beckman Coulter DTX 880 Multimode Detector plate reader (Analytical Instruments, Golden Valley, MN) at the wavelength of 595 nm.

### Statistical analysis

Differences among treatment groups were analyzed using analysis of variance (ANOVA). Differences in which *p* was less than 0.05 were considered statistically significant. In cases where significant differences were detected, specific *post-hoc* comparisons between treatment groups were examined with Student-Newman-Keuls tests. The prevalence of metastasis between control and ethanol-treated groups was determined by the Fisher exact test.

## Results

### PFD inhibits p38γ MAPK activation in breast cancer cells *in vitro* and *in vivo*

**Figure 1:**
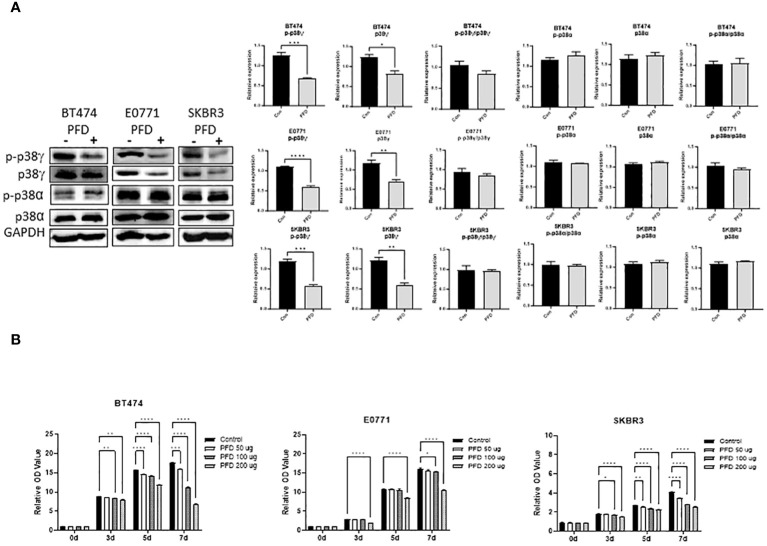
Effects of PFD on the phosphorylation of p38γ MAPK and the growth of breast cancer cells *in vitro.* **(A)** PFD specifically inhibited p38γ MAPK phosphorylation and expression in cultured breast cell lines. Mouse breast cancer cells (E0771) and human breast cancer cell lines (SKBR3 and BT474) were treated with PFD at 200 µg/ml in DMSO for three days. The controls received equal amount of DMSO only. Cell lysates were collected and subjected to immunoblotting (IB) analysis of the phosphorylation of p38γ and p38α MAPK (left panel). The expression of phosphorylated p38γ and p38α MAPK was quantified and normalized to the levels of GAPDH, p38γ or p38α MAPK, respectively (right panel). * p < 0.05, ** p < 0.01, *** p < 0.001 or **** p < 0.0001 denotes significant difference from controls. Each data point was the mean ± SEM of three independent experiments. **(B)** PFD inhibited growth of cultured breast cell lines. E0771, SKBR3 and BT474 cells were treated with PFD at 50, 100 or 200 µg/ml for 3-7 days (3-7d). The cell viability was determined by MTT assay as described in Materials and Methods. * p < 0.05, ** p < 0.01, *** p < 0.001, **** p < 0.0001 denotes significant difference from controls. Each data point was the mean ± SEM of three independent experiments.

**Figure 2:**
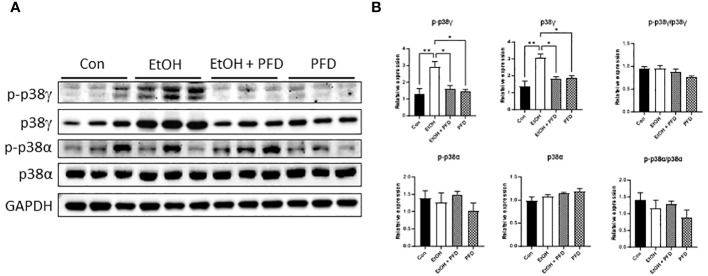
Effects of PFD on alcohol-induced expression and phosphorylation p38γ MAPK in mammary tissues of MMTV-Wnt1 mice. Adolescent MMTV-Wnt1 mice (5-weeks-old) were exposed to alcohol through liquid diet as described in Materials and Methods. PFD (100 mg/kg in DMSO) was delivered by IP injection every other day starting two days before alcohol exposure is initiated. The controls received IP injection of equal amount of DMSO. Mice were euthanized when tumors reached 20 mm maximum diameter. **(A)** Mammary tissues were collected and processed for IB analysis of phosphorylation/expression of p38γ and p38α MAPK. **(B)** The expression of phosphorylated and total p38γ and p38α MAPK was quantified and normalized to the levels of GAPDH, p38γ or p38α MAPK, respectively. *denotes significant difference from controls, * p < 0.05, ** p < 0.01, n = 3.

It was reported that PFD is a pharmacological inhibitor of p38γ MAPK (14–16). We first wanted to determine whether PFD inhibited p38γ MAPK activation in breast cancer cells *in vitro* and *in vivo*. As shown in **Figure 1A**, PFD effectively decreased the phosphorylation and the expression of p38γ MAPK in cultured mouse and human breast cancer cells without affecting other p38 MAPK isoform (p38α MAPK). Consistently, PFD inhibited the growth of these breast cancer cells (**Figure 1B**). To test whether PFD was effective *in vivo*, we IP injected PFD into adolescent MMTV-Wnt1 mice. We demonstrated that PFD administration inhibited alcohol-induced expression and phosphorylation of p38γ MAPK in the mammary tissues of MMTV-Wnt1 mice without affecting p38α MAPK (**Figure 2**).

### PFD inhibits mammary tumor growth and alcohol-stimulated metastasis

**Figure 3:**
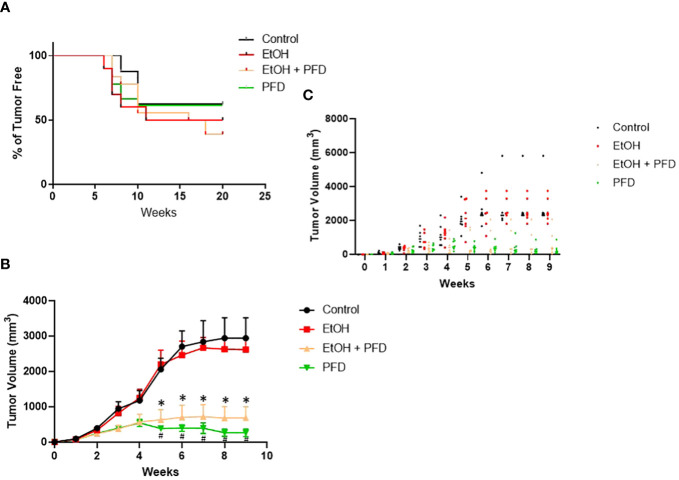
Effects of PFD on the onset of tumorigenesis and growth of mammary tumors. **(A)** Adolescent MMTV-Wnt1 mice were exposed to alcohol through liquid diet as described above. PFD (100 mg/kg) was delivered by IP injection every other day starting two days before alcohol exposure is initiated. The mice were monitored weekly for the appearance and growth of mammary tumors. The percentage of tumor-free mice was determined. n = 16 for control group; n = 20 for alcohol-exposed group (EtOH); n = 18 for the PFD; n = 18 for the PFD + EtOH group. **(B)** Adolescent MMTV-Wnt1 mice were exposed to alcohol and PFD as described above. The tumor volume was measured weekly as described in Materials and Methods. The average was calculated from tumor bearing mice only. # denote significant difference between control and PFD group; * denote significant difference between EtOH and EtOH + PFD group, p < 0.05; n = 6 for control group; n = 6 for EtOH group; n = 7 for PFD group; n = 6 for EtOH + PFD group. **(C)** Individual data points on tumor volumes are shown.

**Figure 4:**
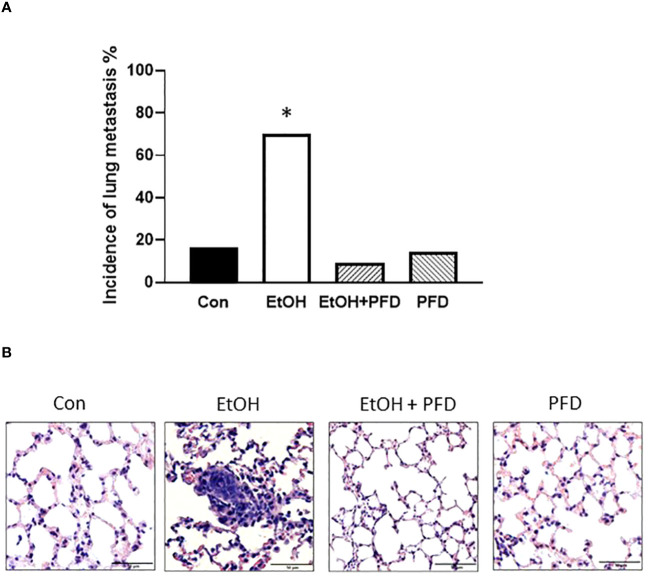
Effects of PFD on metastasis of breast cancer cells *in vivo.* Adolescent MMTV-Wnt1 mice were exposed to alcohol and PFD as described above. When tumor size reached maximal diameter of 20 mm, the mice were euthanized, and the lung metastasis was determined **(A)**. A representative image of H&E staining of lung metastasis from each group was shown **(B)**. * denote significant difference from other groups, p < 0.05; n = 6 for control group; n = 10 for EtOH group; n = 7 for PFD group; n = 11 for EtOH + PFD group. The incidence of lung metastases: Control: 1 out of 6; EtOH: 7 out of 10; PFD: 1 out of 7; EtOH + PFD: 1 out of 11.

Our previous studied indicated that p38γ MAPK activation played an important role in alcohol-induced metastasis of breast cancer (7–9). We sought to determine whether PFD was able to inhibit alcohol-induced promotion of breast cancer. As shown in **Figure 3A**, PFD had little effect on the onset of mammary tumorigenesis. Alcohol did not significantly enhance the growth rate of mammary tumors but drastically promoted metastasis in the lung (**Figures 3B**, **4**). PFD treatment completely abolished the growth of mammary tumors (**Figure 3B**). Furthermore, PFD eliminated alcohol-induced metastasis in the lung (**Figure 4**).

### PFD inhibits alcohol-induced increase of tumorsphere formation and cancer stem cell population

**Figure 5:**
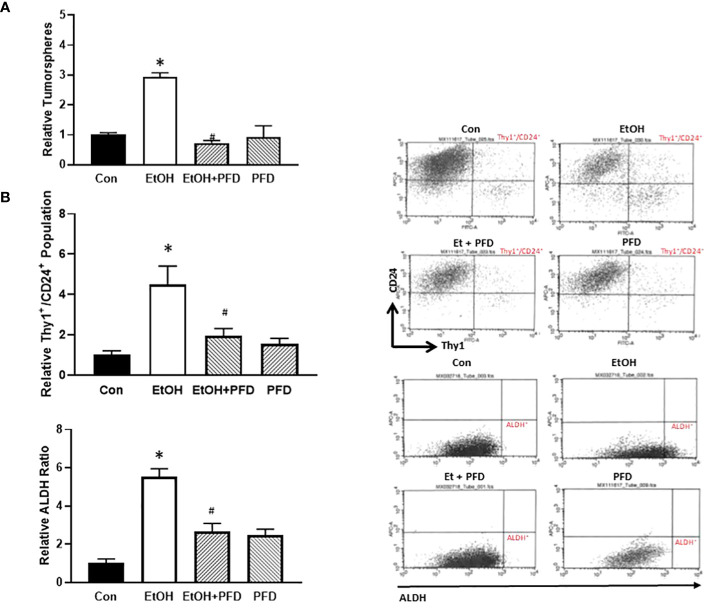
Effects of PFD on the formation of tumorsphere and cancer stem cell (CSC) population. **(A)** Adolescent MMTV-Wnt1 mice were exposed to alcohol and PFD as described above. Mice were euthanized when tumors reached 20 mm maximum diameter, mammary tumor tissues dissected and assayed for tumorsphere formation as described in Materials and Methods. *denotes significant difference from control group, p < 0.05. # denotes significant difference from EtOH group, p < 0.05. n = 6 for control group; n = 10 for EtOH group; n = 7 for PFD group; n = 6 for EtOH + PFD group. **(B)** Adolescent MMTV-Wnt1 mice were exposed to alcohol and PFD as described above. Mice were euthanized when tumors reached 20 mm maximum diameter, and mammary tumor tissues dissected, and tumor cells were isolated. The CSC population in tumor cells was determined and calculated by flow cytometry analysis of Thy1+/CD24+ (top panel) and ALDH ratio (bottom panel) as described in Materials and Methods. Representative FACS plots of Thy1/CD24 and ALDH staining were shown on the left. * denotes significant difference from controls p < 0.05; # denotes significant difference from alcohol-treated group, p < 0.05, n = 6 for control group; n = 10 for EtOH group; n = 7 for PFD group; n = 6 for EtOH + PFD group.

Tumorsphere formation and CSC population are indicative of the aggressiveness of breast cancer (10). Our previous studied suggested that alcohol-induced activation of p38γ MAPK may be involved in regulating tumorsphere formation and CSC population (7–9). Therefore, we sought to determine whether PFD could inhibit alcohol-induced formation of tumorspheres and CSCs. As shown in **Figure 5A**, PFD indeed blocked alcohol-stimulated formation of tumorspheres. Using two CSC assays (ALDH activity and Thy1+/CD24+ population), we showed that PFD significantly inhibited on alcohol-stimulated CSC population (**Figure 5B**). These data are consistent with the findings that PFD can block alcohol-promoted aggressiveness of breast cancer (**Figures 3**, **4**).

## Discussion

We used MMTV-Wnt1 mice to evaluate the effects of PFD on alcohol-induced tumor promotion. Transgenic expression of Wnt1 using a mouse mammary tumor virus LTR enhancer causes extensive ductal hyperplasia early in life and mammary adenocarcinomas in approximately 50% of the female transgenic (MMTV-Wnt1) mice by 6 months of age (24). In this animal model, metastasis to the lung and proximal lymph nodes is rare at the time tumors are detected but may occur at the later times (24). In our study, metastasis to the lung and proximal lymph nodes was rarely observed in the absence of alcohol exposure. This study was the first to determine the effects of PFD, an inhibitor of p38γ MAPK, on alcohol-induced promotion of breast cancer. We demonstrated here PFD treatment blocked alcohol-promoted tumor growth and metastasis in adolescent MMTV-Wnt1 mice. It also significantly inhibited alcohol-induced tumorsphere formation and CSC population. Thus, PFD may be beneficial in treating aggressive breast cancer and particularly effective for ameliorating alcohol-promoted progression of breast cancer. Since PFD is an FDA-approved drug, our findings have important implication for repurposing this drug.

Drug repurposing strategy is to identify new clinical applications of drugs that are already approved for the treatment of other medical conditions. This innovative process has several advantages, as it reduces or eliminates the steps associated with early pharmacological development, such as safety, toxicity, pharmacokinetic and pharmacodynamic studies, and therefore significantly reduces the time and costs associated with traditional new drug discovery/development. PFD, an anti-fibrotic, anti-inflammatory and antioxidant drug, is approved by the European Medicines Agency (EMA) and the United States Food and Drug Administration (FDA) for the treatment of idiopathic pulmonary fibrosis. It has a wide range of targets due to its ability to diffuse across membranes and is rapidly absorbed from the gastrointestinal tract (25). The main action of PFD is considered as an antagonist of TGF-β signaling. It also has anti-inflammatory effects through suppressing proinflammatory cytokines such as TGF-β, tumor necrosis factor α (TNF-α), interleukin-1 (IL-1), interleukin-6 (IL-6), and several other cytokines (13). It was reported that PFD can attenuate oxidative stress (13). Due to this variety of potential targets, PFD is a valuable candidate for treating a wide range of diseases.

PFD has been tested in preclinical models and clinical trials to treat several cancers including pancreatic cancer, lung cancer, colorectal cancer, liver cancer, renal cancer and breast cancer, and the outcomes are promising (26–30). It appears that the anti-cancer property of PFD involves in different mechanisms. For example, the anti-colorectal cancer property seems mediated by PFD’s effects on TGF-β signaling. PFD inhibited TGF-β-induced cell proliferation, migration, and tumor progression of colorectal cancer (25). PFD blocked alcohol-stimulated TGF-β signaling and cell migration/invasion and the epithelial-mesenchymal transition (EMT) in cultured colorectal cells (31). Similarly, PFD suppressed the metastasis triple-negative breast cancer by inhibiting TGF-β/SMAD pathway (20). PFD may inhibit cancers by mechanisms other than blocking TGF-β signaling. For instance, PFD is reported to target the tumor microenvironment and tumor-stroma interaction as a *novel* treatment for non-small cell lung cancer (NSCLC) (32). PFD can sensitize NSCLC cells to chemotherapy (33). PFD promoted miR200 expression to down-regulate ZEB1 and repress the EMT of NSCLC (34, 35). PFD attenuated cell proliferation and promoted apoptosis of hepatocellular carcinoma cells by Inhibiting Wnt/β-Catenin signaling pathway (27).

p38γ MAPK has been shown to regulate cell cycle transition, cell mobility, metastasis, EMT, CSC population, and tumorigenesis (10, 36–39). Importantly, p38γ MAPK is overexpressed and implicated in several types of cancers including colorectal cancer, liver cancer, pancreatic cancer, and breast cancer (16, 37, 40, 41); increased p38γ MAPK expression predicts a poor clinical prognosis (18, 36). CSCs are a subpopulation of tumor cells capable of self-renewal and differentiation and involved in tumor initiation, recurrence, progression, chemoresistance and metastasis (38). Overexpression of p38γ MAPK increases CSCs and tumorspheres (38), suggesting that p38γ MAPK may regulate CSC population and cancer aggressiveness. Therefore, targeting p38γ MAPK signaling network could be an important strategy for therapeutic intervention of cancers (39). PFD is identified as a pharmacological inhibitor of p38γ MAPK (14–16). As a result, PFD has been tested for its therapeutic effects in several cancers associated p38γ MAPK activation, such as colorectal cancer, pancreatic cancer and breast cancer and demonstrated effective in inhibiting the tumorigenesis and progression (16, 18, 40, 42). Our findings that PFD can block alcohol-induced growth, CSC population, and metastasis of mammary tumors in mice support the notion that PFD may be beneficial in treating breast cancer, particularly for those of aggressive types and in the context of alcohol exposure. These results may also provide new value for PFD for the treatment of other alcohol-associate diseases. For example, alcohol is a known etiological factor for pancreatitis and pancreatic cancer (43). As a result, PFD may be a candidate drug for treating alcoholic pancreatitis and associated pancreatic cancer. Indeed, PFD is effective ameliorating chronic pancreatitis in mice (44), and inhibiting pancreatic tumorigenesis (16).

Due to the regulatory role of p38γ MAPK in tumorigenesis and progression, development of more specific p38γ MAPK inhibitors other than PFD becomes an important and exciting future research direction. There are some p38γ MAPK inhibitors with varied efficacy and specificity (39). Recently, some *novel* p38γ MAPK inhibitors with higher specificity are developed (41, 45). One of them, CSH71 which targets the lipid-binding-domain (LBD) of p38γ MAPK shows high specificity. CSH71 is selectively cytotoxic to cutaneous T-cell lymphoma (CTCL) Hut78 cells but spares normal healthy peripheral blood mononuclear (PBMC) cells (41). These *novel* p38γ MAPK inhibitors are promising for the treatment of alcohol-stimulated cancers and other diseases. It is unclear how alcohol activates p38γ MAPK. It has been proposed that the interaction of several upstream signaling molecules, such as CD40L and CD40, may result in p38 MAPK activation which stimulates diverse cytokine profile, transcription factors and oxidative stress (46). These processes may be involved metastasis or cancer stemness. Therefore, understanding of these signaling cascades may be helpful to develop additional targets for the treatment of alcohol-induced tumor promotion.

## Data availability statement

The original contributions presented in the study are included in the article/**Supplementary Material**. Further inquiries can be directed to the corresponding author.

## Ethics statement

Ethical approval was not required for the studies on humans in accordance with the local legislation and institutional requirements because only commercially available established cell lines were used. The animal study was approved by Institutional Animal Care and Use Committee (IACUC) of the University of Kentucky and the University of Iowa. The study was conducted in accordance with the local legislation and institutional requirements.

## Author contributions

HL: Data curation, Formal analysis, Investigation, Methodology, Writing – review & editing. MX: Conceptualization, Data curation, Formal analysis, Investigation, Methodology, Writing – review & editing. DC: Data curation, Investigation, Methodology, Writing – review & editing. WW: Methodology, Writing – review & editing. JL: Conceptualization, Funding acquisition, Investigation, Project administration, Resources, Supervision, Writing – original draft, Writing – review & editing.

## Supplementary material

Supplementary Figure 1 Gross image of the lung showing potential metastases. Mice were treated as described in. The lungs were dissected, and gross images were recorded. Arrow indicates a potential lung tumor.
