# Supplementary figures and images for: Pirfenidone ameliorates alcohol-induced promotion of breast cancer in mice
Source: Front Oncol. 2024 Mar 25;14:1351839. doi: 10.3389/fonc.2024.1351839 (PMC10999600; doi:10.3389/fonc.2024.1351839)

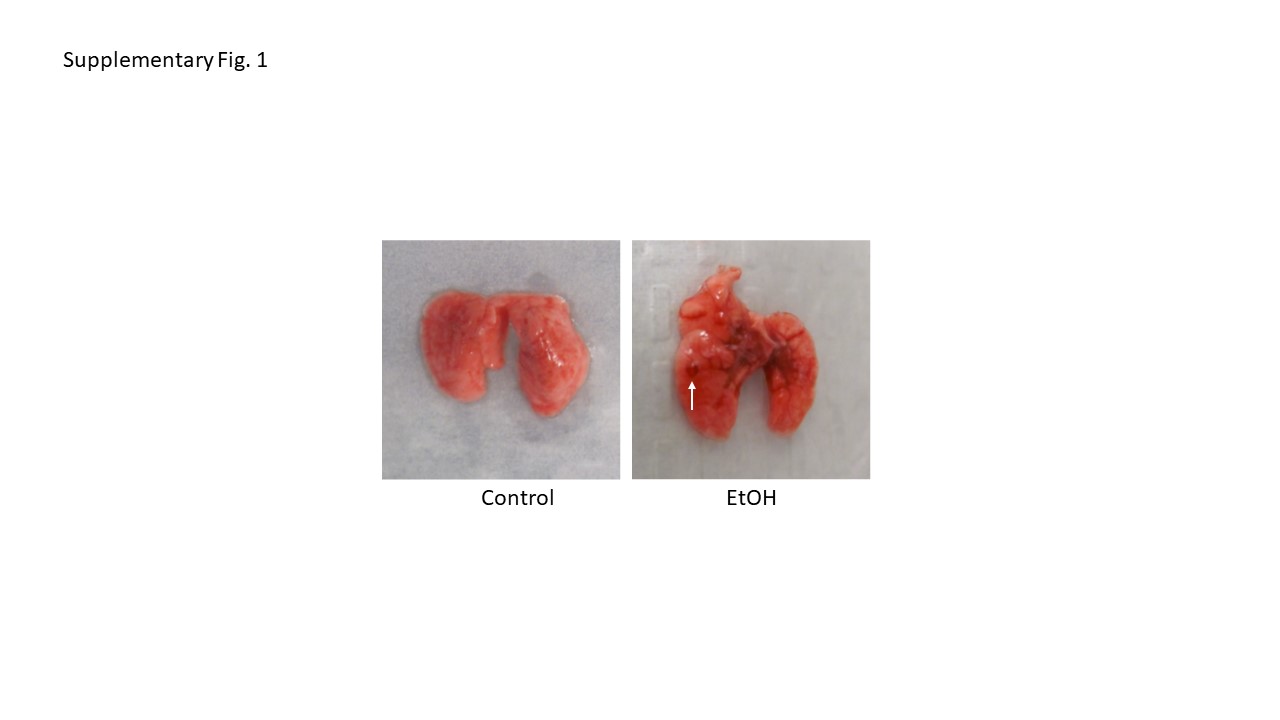

Supplement: Supplementary Figure 1 — Gross image of the lung showing potential metastases. Mice were treated as described in. The lungs were dissected, and gross images were recorded. Arrow indicates a potential lung tumor. [file Image_1.jpeg]
